# Evaluation of Disease Complications Among Adults With Type 1 Diabetes and a Family History of Type 2 Diabetes in Taiwan

**DOI:** 10.1001/jamanetworkopen.2021.38775

**Published:** 2021-12-14

**Authors:** Chia-Hung Lin, Fu-Sung Lo, Yu-Yao Huang, Jui-Hung Sun, Szu-Tah Chen, Chang-Fu Kuo, Mei-Yun Hsieh, Sheng-Hwu Hsieh

**Affiliations:** 1 Division of Endocrinology and Metabolism, Department of Internal Medicine, Chang Gung Memorial Hospital, Linkou, Taiwan; 2 Department of Chinese Medicine, College of Medicine, Chang Gung University, Taoyuan, Taiwan; 3 Division of Pediatric Endocrinology and Genetics, Department of Pediatrics, Chang Gung Memorial Hospital, Linkou, Taiwan; 4 Division of Rheumatology, Allergy and Immunology, Chang Gung Memorial Hospital, Linkou, Taiwan; 5 Division of Rheumatology, Orthopaedics, and Dermatology, School of Medicine, University of Nottingham, Nottingham, United Kingdom

## Abstract

**Importance:**

Patients with type 1 diabetes (T1D) and a family history of type 2 diabetes (T2D) appear to be at a high risk of diabetes complications and other cardiovascular diseases. However, estimates of individual risks in patients in Taiwan are largely unavailable or unreliable.

**Objective:**

To evaluate the risk of diabetes complications and major adverse cardiovascular events (MACEs) in patients with T1D with a family history of T2D.

**Design, Setting, and Participants:**

A population-based cohort study used the Taiwan National Health Insurance Research Database. Participants included all individuals registered in that database on December 31, 2017, and followed up since March 1, 1995. The data were analyzed from December 6, 2018, to December 5, 2019.

**Exposure:**

Patients with T1D and a family history of T2D were evaluated.

**Main Outcomes and Measures:**

The prevalence and hazard ratios (HRs) of diabetes complications and other cardiovascular diseases in patients with T1D were analyzed. The MACEs were identified by diagnostic or procedural codes and heritability was formulated by the registry data of beneficiaries.

**Results:**

Of 27 370 965 individuals included in the database, 11 237 (mean [SD] age, 22.7 [14.4] years; 54% were female) had T1D. The crude prevalence of T1D was 0.04%, with a female to male ratio of 1.22: 1. The adjusted HRs in individuals who had a first-degree relative with T2D were 2.61 (95% CI, 1.32-5.16) for MACEs at an age at diagnosis of less than 20 years. Adjusted HRs were 1.44 (95% CI, 1.27-1.64) for diabetic neuropathy, 1.28 (95% CI, 1.12-1.47) for retinopathy, and 1.24 (95% CI, 1.06-1.47) for neuropathy at all ages of diagnosis.

**Conclusions and Relevance:**

In this study of patients in Taiwan with T1D, having relatives with T2D was associated with an increase in the individual risks of developing diabetes complications. Patients with T1D and a family history of T2D might have more complications and require close management.

## Key Points

**Question:**

Is a family history of type 2 diabetes associated with outcomes among adults with type 1 diabetes in Taiwan?

**Findings:**

In this cohort study of 11 237 patients with type 1 diabetes, the hazard ratios of major adverse cardiovascular events for patients with a family history of type 2 diabetes were higher than for those without a family history at age at diagnosis of less than 20 years. The risk of diabetic nephropathy, retinopathy, and neuropathy was also higher for patients who had a first-degree relative with type 2 diabetes after multiple adjustments at all ages of diagnosis.

**Meaning:**

In this study, patients with type 1 diabetes and a family history of type 2 diabetes had more complications than those without a family history of type 2 diabetes.

## Teaser

This cohort study assesses whether a positive family history of presumed type 2 diabetes is associated with the microvascular and macrovascular complications of type 1 diabetes in a population of adults in Taiwan.

## Introduction

Both type 1 diabetes (T1D) and type 2 diabetes (T2D) are associated with an increased risk of major adverse cardiovascular events (MACEs).^1^ Type 1 diabetes is an autoimmune disease that causes destruction of β cells in the pancreas, resulting in an absolute insulin deficiency.^2^ Type 2 diabetes is associated with insulin resistance leading to hyperinsulinemia, α-cell dysfunction, hyperglycemia, and eventually insulin deficiency.^2^ Type 1 diabetes and T2D differ in pathophysiologic characteristics and risk factors for cardiovascular disease. The MACEs seen in T2D are associated with insulin resistance,^3^ whereas those in T1D are associated with the presence of renal disease.^4^ However, whether the cardiovascular risk in T1D was associated with the presence of a positive T2D family history is largely unknown. Because these 2 diseases have different mechanisms, it may be possible that both diseases could develop in a certain proportion of individuals.

The coexistence of spontaneous insulin deficiency and inherited insulin resistance could therefore be considered to be a risk factor for cardiovascular disease, which would likely lead to an increased risk for MACEs. To explore the possibility that individuals with both T1D and T2D are at a higher risk for MACEs, a positive family history of T2D has been used as a marker for susceptibility to MACEs in individuals with T1D.^5^ Moreover, first-degree relatives of individuals with T2D have been reported to be hyperinsulinemic and to have a more atherogenic pattern of cardiovascular risk factors.^6^ The data regarding T2D family history and the outcomes of T1D in the Taiwan population are limited. Therefore, the aim of this study was to examine the association between a positive family history of presumed T2D and the microvascular and macrovascular complications of T1D in a population from a nationwide Taiwan database.

## Methods

### Study Population

Since 1995, the Taiwan National Health Insurance (NHI) Research Database (NHIRD) has recorded the sex, date of birth, place of residence, details of insurance (including employment category, amount of insurance paid, enrollment, and discharge date), family relationships, vital statistics, and details of clinical information (including dates of inpatient and outpatient visits, medical diagnoses, medical expenditures, prescription details, vaccination status, examinations, operations, and procedures) of its population. The NHI coverage rate was more than 99.9% of the population of Taiwan in 2015.^7^ The National Health Research Institute acquires all data from the Department of Health and Welfare and compiles them into an electronic database, the NHIRD. All of the information is linked using a unique personal identification number assigned to each resident in Taiwan. To ensure confidentiality, this number is encrypted before the data are released to researchers; however, the identification number remains unique for each beneficiary in the database to facilitate internal linkage of records.

The study population included 27 370 965 individuals enrolled in the NHI program in Taiwan from March 1, 1995, to December 31, 2017. The data were analyzed from December 6, 2018, to December 5, 2019. We established a cohort of all individuals registered in the NHIRD in 2017 using data from the registry for NHI beneficiaries, a registry for patients with a catastrophic illness, and data sets of ambulatory care expenditures and details of ambulatory case orders, all of which are included in the NHIRD. Individuals without valid insurance status were excluded from analysis. This study was approved by the Institutional Review Board of Chang Gung Memorial Hospital, Linkou, Taiwan. The need for informed consent was waived for this study because all data in the NHIRD are anonymous. This study followed the Strengthening the Reporting of Observational Studies in Epidemiology (STROBE) reporting guideline for cohort studies.

Methods to identify first-degree relatives in the NHIRD have been reported.^8^ In brief, the registry of beneficiaries contains identifiers of the relationships between the insured person (who paid the insurance fee) and his or her dependents. Only blood relatives and spouses are eligible to be claimed as dependents of an insured person. A birth certificate is used by the medical facility that delivered the child, or a DNA parentage test for those who were not born in medical facilities is required for a child to be registered as a dependent of the parents. We could therefore establish family relationships using these identifiers and unique personal identification of parents, grandparents, children, grandchildren, and spouses. In general, parent-offspring relationships and spouses could be identified directly. An algorithm allowing indirect identification of parent-offspring relationships was also used to assess possible family links.^8^ Full siblings of an individual were identified if they had the same parents. Twins were identified as full siblings with the same date of birth (±1 day). However, twin zygosity cannot be derived from the database. For analysis among people from the same family, individuals were grouped into families according to their relationships.

Among 27 370 965 beneficiaries in the NHI (both alive and dead between March 1, 1995. and December 31, 2017), 6 925 954 individuals were registered alone without any identifiable relative. The other 20 445 011 individuals were classified into 4 145 976 families. Overall, 21 767 880 parent-child relationships, 16 245 232 full sibling pairs, and 260 844 twin pairs were identified. Each individual may have appeared multiple times in different categories of family relationships depending on the family structure.

### Ascertainment of Type 1 Diabetes

In Taiwan, patients with suspected T1D are referred to specialists for diagnosis and treatment. Patients with a diagnosis of T1D included in this study were exempt from medical copayments. Diagnostic information including examination results, fasting or glucagon-stimulated C-peptide level, anti–glutamic acid decarboxylas antibody level, and history of diabetic ketoacidosis is sent to the insurance administration for review by commissioned expert panels to confirm the diagnosis before approval of the exemption. A diagnosis of T1D in the Catastrophic Illness Database has been used to report the incidence of T1D in Taiwan with a positive predictive rate of 98.3%.^9^

The Registry for Catastrophic Illness Patients^10^ contains information on these patients with unique personal identification codes, diagnosis, demographic characteristics, application date, diagnosing physician, hospital, and other administration data. We used this registry to identify patients with T1D.

### Covariates

We considered age, sex, occupation category, income level quintile, level of urbanization of residence, and family size. These covariates may have confounded or modified the familial associations.^8^

### Outcomes Measurement

MACES were identified by *International Classification of Diseases, Ninth Revision (ICD-9)* and *International Statistical Classification of Diseases and Related Health Problems, Tenth Revision (ICD-10)* codes including myocardial infarction, heart failure, stroke, malignant dysrhythmia, and cardiogenic shock or by procedural codes used by the Taiwan NHI including percutaneous coronary intervention, coronary artery bypass surgery, and thrombolysis therapy. The other complication of T1D was also defined by *ICD* codes (eTable 1 in the Supplement).

### Statistical Analysis

Heritability is defined as the proportion of phenotypic variance attributable to genetic factors, and the definition of familial transmission is the proportion of genetic and shared environmental contributions. Familial transmission and heritability can be calculated using the polygenic liability model to calculate both measures.^11,12,13^ This model assumes a normally distributed liability of disease resulting from small and additive influences from a large number of unspecified genes and environmental factors. The liability of the affected individuals is greater than a critical threshold, the value of which can be determined using information on disease prevalence in the affected individuals and general population.

In the present study, familial transmission was defined as the function of the difference in normal variance of the threshold from the mean liability between individuals with affected relatives and the healthy population.^8,14^ The original model assumes no common environmental variance; therefore, familial transmission equals heritability. To account for shared environmental factors in phenotypic variance, we used the spouse as a control, assuming that spouses share the family environment but have no close genetic similarity with blood-related family members. We restricted a family history to first-degree relatives and assumed a mean of 2 siblings in a family.

An alternative method to estimate heritability is based on comparing tetrachoric correlations that are used as an index of phenotypic similarity between siblings and spouses, assuming that they have a similar shared environment but 50% and 0% genetic similarity. We used this method to calculate heritability as 2 × (tetrachoric correlation for full siblings − tetrachoric correlation for spouse).

The baseline characteristics of the 2 groups were compared using analysis of variance for continuous variables and the χ^2^ test for categorical variables. In the multivariate analysis, logistic regression models were adjusted for confounding factors including age, sex, hypertension, hyperlipidemia, nephropathy, retinopathy, and peripheral neuropathy. Associations between a family history of T2D and MACEs in the patients with T1D were analyzed using a Cox proportional hazards regression model, and the results are presented as unadjusted hazard ratios (HRs) with 95% CIs. For each of the 2 comparisons, we used separate logistic regression models to estimate a propensity score and then created matched pairs. The characteristics and Cox proportional hazards regression models for adjusted HRs and 95% CIs are also presented for the matched pairs. The analysis was also performed by subgroups according to sex and age. All tests of statistical hypotheses were performed at a 2-sided *P* = .05 level of significance. All analyses were performed using SAS, version 9.3 (SAS Institute Inc).

## Results

A total of 11 237 patients (mean [SD] age, 22.7 [14.4] years; 54% were female) had a diagnosis of T1D, with a crude prevalence of 0.04%. The mean (SD) time of evolution from the diagnosis of T1D to complication identified was 8.59 (1.37) years. Women had a higher prevalence (0.05%) than men (0.04%), with a female to male ratio of 1.22: 1 (Table 1). In the general population of Taiwan in 2017, a total of 1302 individuals (11.58%) with T1D had at least 1 first-degree relative with T2D.

**Table 1.** Demographic Data of Patients With T1D

Characteristic	Male patients, No. (%)		*P* value	Female patients, No. (%)		*P* value
	T2D family history			T2D family history		
	With (n = 600)	Without (n = 4572)		With (n = 702)	Without (n = 5363)	
Age, mean (SD), y	25.07 (12.37)	22.39 (14.65)	<.001	21.87 (10.51)	22.76 (14.83)	.04
Age, y						
<5	14 (2.33)	386 (8.44)	<.001	13 (1.85)	368 (6.86)	<.001
5-9	26 (4.33)	585 (12.80)		48 (6.84)	739 (13.78)	
10-14	105 (17.50)	842 (18.42)		145 (20.66)	911 (16.99)	
15-19	95 (15.83)	544 (11.90)		143 (20.37)	727 (13.56)	
20-24	81 (13.50)	471 (10.30)		109 (15.53)	587 (10.95)	
25-29	82 (13.67)	426 (9.32)		96 (13.68)	539 (10.05)	
≥30	197 (32.83)	1318 (28.83)		148 (21.08)	1492 (27.82)	
Place of residence						
Urban	341 (56.83)	2765 (60.48)	.27	425 (60.54)	3358 (62.61)	.24
Suburban	202 (33.67)	1361 (29.77)		214 (30.48)	1548 (28.86)	
Rural	46 (7.67)	353 (7.72)		46 (6.55)	375 (6.99)	
Unknown	11 (1.83)	93 (2.03)		17 (2.42)	82 (1.53)	
Income levels						
Quintile 1	134 (22.33)	865 (18.92)	.30	138 (19.66)	967 (18.03)	.14
Quintile 2	77 (12.83)	646 (14.13)		125 (17.81)	829 (15.46)	
Quintile 3	154 (25.67)	1146 (25.07)		172 (24.50)	1410 (26.29)	
Quintile 4	112 (18.67)	852 (18.64)		131 (18.66)	1036 (19.32)	
Quintile 5	113 (18.83)	993 (21.72)		123 (17.52)	1061 (19.78)	
Unknown	10 (1.67)	70 (1.53)		13 (1.85)	60 (1.12)	
Occupation						
Dependents of the insured individuals	274 (45.67)	2619 (57.28)	<.001	399 (56.84)	3290 (61.35)	.01
Civil servants, teachers, military personnel, and veterans	9 (1.50)	102 (2.23)		10 (1.42)	96 (1.79)	
Nonmanual workers and professionals	146 (24.33)	834 (18.24)		155 (22.08)	890 (16.60)	
Manual workers	91 (15.17)	524 (11.46)		74 (10.54)	638 (11.90)	
Other	80 (13.33)	493 (10.78)		64 (9.12)	449 (8.37)	
Comorbidity						
Myocardial infarct	3 (0.50)	27 (0.59)	>.99	NR^a^	26 (0.48)	.36
Congestive heart failure	19 (3.17)	96 (2.10)	.10	14 (1.99)	117 (2.18)	.75
Peripheral vascular disease	17 (2.83)	133 (2.91)	.92	13 (1.85)	115 (2.14)	.61
Cerebrovascular disease	16 (2.67)	174 (3.81)	.16	22 (3.13)	204 (3.80)	.38
Dementia	NR^a^	26 (0.57)	.77	3 (0.43)	17 (0.32)	.50
Chronic pulmonary disease	64 (10.67)	658 (14.39)	.01	82 (11.68)	713 (13.29)	.23
Rheumatologic disease	6 (1.00)	58 (1.27)	.58	22 (3.13)	201 (3.75)	.42
Ulcer disease	97 (16.17)	643 (14.06)	.17	107 (15.24)	892 (16.63)	.35
Hemiplegia	7 (1.17)	17 (0.37)	.02	4 (0.57)	23 (0.43)	.60
Mild liver disease	42 (7.00)	312 (6.82)	.87	21 (2.99)	210 (3.92)	.23
Moderate or severe liver disease	NR^a^	4 (0.09)	.46	0	3 (0.06)	NA
Diabetes with end-organ damage	300 (50.00)	2116 (46.28)	.09	337 (48.01)	2616 (48.78)	.70
Any tumor	15 (2.50)	85 (1.86)	.28	17 (2.42)	146 (2.72)	.64
Metastatic solid tumor	0	NR^a^	NA	0	NR^a^	NA
AIDS	3 (0.50)	8 (0.17)	.13	0	0	NA

Abbreviations: NA, not applicable; NR, not reported; T1D, type 1 diabetes; T2D, type 2 diabetes.

^a^ Numbers <3 are not displayed, as per the confidentiality policies of the National Health Insurance Database.

Among the age distribution, the proportion of those with a younger age (<10 years) was higher in the patients without a family history of T2D (20.92% vs 7.76 %; *P* < .001). The place of residence and income level were not relevant to the status of family history of T2D. With regard to comorbidities, men without a family history of T2D had a higher rate of pulmonary disease than those with a family history of T2D (14.39% vs 10.67%; *P* = .01) (Table 1).

The adjusted HR for MACEs was 1.15 (95% CI, 0.82-1.59) in the patients with a history of T2D, although these data were not statistically significant (Table 2). For diabetic nephropathy, the adjusted HR was higher at 1.44 (95% CI, 1.27-1.64), for retinopathy, 1.28 (95% CI, 1.12-1.47), and for peripheral neuropathy, 1.24 (95% CI, 1.06-1.47). Subgroup analysis based on sex showed that the adjusted HR decreased to 1.37 (95% CI, 1.16-1.63) in the female group for diabetic nephropathy (*P* < .05 for interaction) (Table 3).

**Table 2.** Outcomes in Patient With T1D According to the Presence of a T2D Family History^a^

Variable	T2D family history, No. (%)		HR (95% CI)		*P* value for interaction of time
	With (n = 1302)	Without (n = 9935)	Crude	Adjusted	
Stroke	13 (1.00)	151 (1.52)	0.79 (0.45-1.40)	0.64 (0.36-1.14)	.04^b^
CAD	6 (0.46)	57 (0.57)	1.03 (0.44-2.39)	0.86 (0.37-2.01)	.26
MACEs	41 (3.15)	292 (2.94)	1.33 (0.96-1.85)	1.15 (0.82-1.59)	.39
Nephropathy	276 (21.20)	1841 (18.53)	1.46 (1.29-1.66)^b^	1.44 (1.27-1.64)^b^	.01^b^
Retinopathy	242 (18.59)	1641 (16.52)	1.39 (1.22-1.59)^b^	1.28 (1.12-1.47)^a^	.02^b^
Peripheral neuropathy	167 (12.83)	1130 (11.37)	1.32 (1.12-1.55)^b^	1.24 (1.06-1.47)^b^	<.001^b^
Hypertension	221 (16.97)	1472 (14.82)	1.43 (1.24-1.65)^b^	1.28 (1.11-1.48)^b^	.09
Hyperlipidemia	363 (27.88)	2652 (26.69)	1.41 (1.27-1.58)^b^	1.36 (1.21-1.51)^b^	<.001^b^

Abbreviations: CAD, coronary artery disease; HR, hazard ratio; MACEs, major adverse cardiovascular events; T1D, type 1 diabetes; T2D, type 2 diabetes.

^a^ Factors were adjusted for dementia, chronic pulmonary disease, rheumatologic disease, ulcer disease, liver disease, and any tumor.

^b^ *P* < .05.

**Table 3.** Outcomes in Patients With T1D According to the Presence of a T2D Family History, by Male and Female Patients^a^

Variable	Male patients				Female patients				P value for interaction of sex
	T2D family history, No. (%)		HR (95% CI)		T2D family history		HR (95% CI)		
	With (n=600)	Without (n=457)	Crude	Adjusted	With (n=702)	Without (n=536)	Crude	Adjusted	
Stroke	6 (1.00)	80 (1.75)	0.68 (0.30-1.56)	0.36 (0.15-0.85)	7 (1.00)	71 (1.32)	0.93 (0.43-2.01)	0.91 (0.42-1.99)	.54
CAD	3 (0.50)	31 (0.68)	0.92 (0.28-3.02)	0.76 (0.23-2.51)	3 (0.43)	26 (0.48)	1.16 (0.35-3.85)	1.08 (0.32-3.60)	.91
MACEs	25 (4.17)	157 (3.43)	1.51 (0.99-2.30)	1.03 (0.67-1.59)	16 (2.80)	135 (2.52)	1.14 (0.68-1.91)	1.12 (0.67-1.89)	.72
Nephropathy	119 (19.83)	777 (16.99)	1.60 (1.32-1.94)^b^	1.51 (1.24-1.83)^b^	157 (22.36)	1064 (19.84)	1.36 (1.15-1.60)^b^	1.37 (1.16-1.63)^b^	<.001^b^
Retinopathy	95 (15.83)	695 (15.20)	1.33 (1.07-1.65)^b^	1.13 (0.91-1.41)	147 (20.94)	946 (17.64)	1.43 (1.20-1.70)^b^	1.40 (1.17-1.66)^b^	<.001^b^
Peripheral neuropathy	72 (12.00)	488 (10.67)	1.36 (1.06-1.75)^b^	1.22 (0.95-1.57)	95 (13.53)	642 (11.97)	1.28 (1.03-1.59)^b^	1.25 (1.01-1.56)^b^	.01^b^
Hypertension	116 (19.33)	732 (16.01)	1.63 (1.34-1.98)^b^	1.38 (1.13-1.69)^b^	105 (14.96)	740 (13.80)	1.28 (1.04-1.57)^b^	1.22 (0.99-1.50)	.23
Hyperlipidemia	146 (24.33)	1152 (25.20)	1.41 (1.18-1.67)^b^	1.32 (1.11-1.57)^b^	217 (30.91)	1500 (27.97)	1.41 (1.22-1.63)^b^	1.37 (1.19-1.58)^b^	<.001^b^

Abbreviations: CAD, coronary artery disease; HR, hazard ratio; MACEs, major adverse cardiovascular events; T1D, type 1 diabetes; T2D, type 2 diabetes.

^a^ Factors were adjusted for dementia, chronic pulmonary disease, rheumatologic disease, ulcer disease, liver disease, and any tumor.

^b^ *P* < .05.

Further stratification of the results by age group is presented in Table 4. The adjusted HR of MACEs for patients with an age at diagnosis of less than 20 years was 2.61 (95% CI, 1.32-5.16), which was higher than for those with an age greater than or equal to 20 years. For the microvascular complications, the adjusted HRs were higher in the group with an age at diagnosis of less than 20 years compared with the group with an ag greater than or equal to 20 years (for nephropathy, HR, 2.06, 95% CI, 1.71-2.49 vs 1.08, 95% CI, 0.91-1.28; for retinopathy, HR, 1.78, 95% CI, 1.47-2.17 vs 0.99, 95% CI, 0.81-1.20; and for peripheral nephropathy, HR, 1.56, 95% CI, 1.19-2.05 vs 1.08, 95% CI, 0.88-1.33). For the risk of hypertension, the adjusted HR for the group with an age at diagnosis of less than 20 years was 1.86 (95% CI, 1.47-2.35) vs the group with an age of 20 years or older (1.09; 95% CI, 0.91-1.31). In the risk of hyperlipidemia, the adjusted HRs for the group with an age of less than 20 years was 1.57 (95% CI, 1.35-1.82) vs the group age with an age of 20 years or older 1.13 (95% CI, 0.96-1.32).

**Table 4.** Outcomes in Patients With T1D According to the Presence of a T2D Family History, by Age at Diagnosis^a^

Variable	Age <20 y				Age ≥20 y				*P* value for interaction of age
	T2D family history, No. (%)		HR (95% CI)		T2D family history		HR (95% CI)		
	With (n = 589)	Without (n = 5102)	Crude	Adjusted	With (n = 713)	Without (n = 4833)	Crude	Adjusted	
Stroke	4 (0.68)	11 (0.22)	3.67 (1.17-11.54)^b^	2.31 (0.66-80)	9 (1.26)	140 (2.90)	0.56 (0.28-1.10)	0.53 (0.27-1.05)	.73
CAD	2 (0.34)	4 (0.08)	6.28 (0.57-69.45)	3.59 (0.17-74.69)	4 (0.56)	53 (1.10)	0.85 (0.34-2.12)	0.90 (0.36-2.25)	.20
MACEs	16 (2.72)	30 (0.59)	5.80 (3.16-10.66)^b^	2.61 (1.32-5.16)^b^	25 (3.51)	262 (5.42)	0.85 (0.56-1.28)	0.89 (0.59-1.35)	.02^b^
Nephropathy	133 (22.58)	666 (13.05)	2.21 (1.83-2.66)^b^	2.06 (1.71-2.49)^b^	143 (20.06)	1175 (24.31)	1.04 (0.88-1.24)	1.08 (0.91-1.28)	<.001^b^
Retinopathy	125 (21.22)	641 (12.56)	2.07 (1.71-2.51)^b^	1.78 (1.47-2.17)^b^	117 (16.41)	1000 (20.69)	0.98 (0.81-1.19)	0.99 (0.81-1.20)	<.001^b^
Peripheral neuropathy	64 (10.87)	345 (6.76)	1.87 (1.43-2.44)^b^	1.56 (1.19-2.05)^b^	103 (14.45)	785 (16.24)	1.05 (0.85-1.28)	1.08 (0.88-1.33)	<.001^b^
Hypertension	91 (15.45)	413 (8.09)	2.47 (1.97-3.10)^a^	1.86 (1.47-2.35)^b^	130 (18.23)	1059 (21.91)	1.02 (0.85-1.23)	1.09 (0.91-1.31)	<.001^b^
Hyperlipidemia	199 (33.79)	1348 (26.42)	1.69 (1.45-1.96)^b^	1.57 (1.35-1.82)^b^	164 (23.00)	1304 (26.98)	1.12 (0.96-1.32)	1.13 (0.96-1.32)	<.001^b^

Abbreviations: CAD, coronary artery disease; HR, hazard ratio; MACEs, major adverse cardiovascular events; T1D, type 1 diabetes; T2D, type 2 diabetes.

^a^ Factors were adjusted for dementia, chronic pulmonary disease, rheumatologic disease, ulcer disease, liver disease, and any tumor.

^b^ *P* < .05.

We analyzed the risk by the stratification of either or both parents with T2D in Table 5. The results suggested that the risk was significantly increased in the group containing both parents with T2D than in the group containing only 1 parent (HR. 1.76; 95% CI, 1.23-2.53 vs 1.41; 95% CI, 1.23-1.62 for nephropathy; HR, 2.66; 95% CI, 1.89-3.74 vs 1.20; 95% CI, 1.04-1.40 for retinopathy; and HR, 1.69; 95% CI, 1.06-2.70 vs 1.25, 95% CI, 1.05-1.49 for peripheral neuropathy).

**Table 5.** Outcomes in Patients With T1D According to the Presence of Either Parent or Both Parents With T2D^a^

Variable	With T2D family history		Without T2D family history (n = 9983) [Reference]	With T2D family history, HR (95% CI)			
	In father and mother (n = 125)	In father or mother (n = 1129)		Crude		Adjusted	
				In father and mother	In father or mother	In father and mother	In father or mother
Stroke	1 (0.80)	12 (1.06)	151 (1.51)	1.41 (0.35-5.70)	0.77 (0.42-1.42)	0.95 (0.24-3.86)	0.64 (0.35-1.19)
CAD	2 (1.60)	4 (0.35)	57 (0.57)	4.04 (0.99-16.6)	0.79 (0.28-2.17)	2.48 (0.6-10.24)	0.71 (0.25-1.96)
MACEs	4 (3.20)	32 (2.83)	297 (2.98)	1.50 (0.56-4.03)	1.17 (0.81-1.68)	0.92 (0.34-2.47)	1.05 (0.73-1.52)
Nephropathy	30 (24.00)	236 (20.90)	1851 (18.54)	2.10 (1.46-3.01)^b^	1.41 (1.23-1.61)^b^	1.76 (1.23-2.53)^b^	1.41 (1.23-1.62)^b^
Retinopathy	34 (27.20)	200 (17.71)	1649 (16.52)	2.76 (1.96-3.87)^b^	1.30 (1.12-1.50)^b^	2.66 (1.89-3.74)^b^	1.20 (1.04-1.40)^b^
Peripheral neuropathy	18 (14.40)	147 (13.02)	1132 (11.34)	1.79 (1.13-2.86)^b^	1.32 (1.11-1.57)^b^	1.69 (1.06-2.70)^b^	1.25 (1.05-1.49)^b^
Hypertension	28 (22.40)	184 (16.30)	1481 (14.84)	2.38 (1.64-3.46)^b^	1.33 (1.14-1.56)^b^	1.86 (1.28-2.70)^b^	1.19 (1.02-1.39)^b^
Hyperlipidemia	38 (30.40)	316 (27.99)	2661 (26.66)	2.02 (1.46-2.78)^b^	1.38 (1.23-1.55)^b^	1.84 (1.34-2.54)^b^	1.32 (1.18-1.49)^b^

Abbreviations: CAD, coronary artery disease; HR, hazard ratio; MACEs, major adverse cardiovascular events; T1D, type 1 diabetes; T2D, type 2 diabetes.

^a^ Factors were adjusted for dementia, chronic pulmonary disease, rheumatologic disease, ulcer disease, liver disease, and any tumor.

^b^ *P* < .05.

With regard to the development of comorbidities over time, we found that the difference between patients with and without T2D family history appeared earlier through follow-up in the hyperlipidemia than that in the hypertension analysis (eFigure 1 in the Supplement). The risks were higher in the individuals with a positive family history of T2D than in those without a family history in the survival analysis (adjusted HR, 1.28, 95% CI, 1.11-1.48; *P* < .001 for hypertension and HR, 1.36; 95% CI, 1.21-1.51, *P* < .001 for hyperlipidemia). The patients with a family history of T2D had a higher risk of nephropathy, retinopathy, and peripheral neuropathy than those without a family history of T2D in the survival graphs (eFigure 2-C in the Supplement). These MACEs were developed later in the years after the diagnosed of T1D compared with microvascular complications (eFigure 3A in the Supplement). In addition, the risk of MACEs was higher in the individuals with a positive family history of T2D than in those without a family history of T2D, although the findings were not statistically significant in the survival analysis. The risks of stroke and coronary artery disease were also not significantly different between the 2 groups in the survival graphs (eFigure 3B and 3C in the Supplement).

## Discussion

The proportion of patients with a first-degree family history of T2D was 11.58% in the Taiwanese population, which was lower than that reported in a 1998 study of a White population (approximately 17%).^5^ The odds ratio of coronary artery disease reported in that study^5^ was 1.45 times higher than those without a T2D family history, but the risk was not statistically significant in our study. The lower proportion of patients with a family history of T2D in an Asian population with T1D may be associated with the nonsignificant difference in the coronary artery disease risk.

The association between a family history of T2D and the risk of MACEs in the patients with T1D was less clear. However, we found that a positive family history of T2D was a risk factor associated with MACEs in individuals with T1D diagnosed at an age of less than 20 years. In addition, the incidence of hypertension and hyperlipidemia was statistically significantly higher than in those without the positive family history of T2D. This finding supports the concept that insulin resistance is associated with the development of MACEs in patients with T1D diagnosed early in life. Of current interest is whether individuals with T1D are at an increased risk of MACEs in the presence of a family history of T2D independently of the typical risk factors for T1D. In our analysis, we reported that a family history of T2D among these patients was a statistically significant factor after adjusting for other covariates. In addition, family history can be used as a surrogate for genetic predisposition, and shared environmental factors in the family also played a role in the reported increased risk.

The proxy measure of insulin resistance (a family history of T2D) does not account for the associations between the genetic and environmental factors of insulin resistance. In addition, cohort studies can only suggest an association between a family history and the presence of MACEs. Further investigations, including prospective analyses, are needed to clarify the association between insulin resistance and MACEs in T1D.

A 1987 observational study^15^ investigated the associations between insulin resistance and macrovascular complications in patients with T1D, in which individuals were included if they did not have obesity and had a duration of diabetes greater than 15 years at commencement of the study. An association between insulin resistance and atherosclerotic disease was reported in this population of individuals who did not have obesity, although no measure of body composition was mentioned. This finding is compatible with our results in that the risks of macrovascular complications were increased in the patients with T1D with a positive T2D family history.

There were substantial differences in microvascular complications, including nephropathy in the patients with T1D, between those with a positive and negative family history of T2D in the present study. A small-scale population study^16^ has reported that a parental history of T2D was associated with a 3-fold risk of diabetic nephropathy after adjustment for other risk factors. In a large US cohort study,^17^ the risk for nephropathy increased 2-fold if a parent had T2D, but the risk did not increase if a parent had T1D. In the present study, the risk of diabetic nephropathy has been found to be statistically significantly increased in individuals with a family history of T2D, which is consistent with MACEs. Diabetic nephropathy and MACEs are closely associated, and patients with diabetic nephropathy are far more likely to die of cardiovascular disease than to progress to end-stage kidney disease.^18^ This finding may help explain why we found a higher risk of diabetic nephropathy in the patients with a positive family history of T2D than other microvascular complications in the patients with T1D.

### Limitations

This study has limitations. First, as an observational cohort study, selection bias was possible. However, the relative small incidence of T1D in the Taiwan population reflects the need for a larger cohort size. Second, the data regarding glycemic control were lacking in this database, although glycemic control may be a confounding factor in cardiovascular outcomes. Third, family history of T1D, cardiovascular disease, or kidney disease were not included in the analysis. However, the statistically significant differences between a positive or negative family history of T2D among patients with T1D still provided the clinical implication (that if the patients with T1D had T2D family history, the risk of diabetic nephropathy was much higher than other microvascular complications) in such a critical issue.

## Conclusions

In this cohort study, a family history of T2D was a significant risk factor for the presence of MACEs and microvascular complications in patients with T1D. Type 1 diabetes and T2D with an accompanying increased risk of MACEs suggest that a family history of T2D may be a useful marker to identify a subgroup of patients with T1D with an increased risk of developing MACEs. Having T1D as well as a family history of T2D was associated with increases in individual risks of hypertension, hyperlipidemia, microvascular complications. and MACEs. An increased focus on this patient population in the prevention of these diabetes complications is warranted in further clinical investigations.

## Notes

Supplement. **eTable.** The ICD Coding of Diseases **eFigure 1.** The Development of Comorbidities Over Time. (A) Hypertension. (B) Hyperlipidemia. **eFigure 2.** The Development of Microvascular Complications Over Time. (A) Nephropathy. (B) Retinopathy. (C) Peripheral neuropathy. **eFigure 3.** The Development of Macrovascular Complications Over Time. (A) MACEs (B) Stroke (C) CAD.
