# Supplementary material for: Evaluation of Disease Complications Among Adults With Type 1 Diabetes and a Family History of Type 2 Diabetes in Taiwan
Source: JAMA Netw Open. 2021 Dec 14;4(12):e2138775. doi: 10.1001/jamanetworkopen.2021.38775 (PMC8672229; doi:10.1001/jamanetworkopen.2021.38775)
Supplement: Supplement. — eTable. The ICD Coding of Diseases eFigure 1. The Development of Comorbidities Over Time. (A) Hypertension. (B) Hyperlipidemia. eFigure 2. The Development of Microvascular Complications Over Time. (A) Nephropathy. (B) Retinopathy. (C) Peripheral neuropathy. eFigure 3. The Development of Macrovascular Complications Over Time. (A) MACEs (B) Stroke (C) CAD. [file jamanetwopen-e2138775-s001.pdf]

Supplemental Online Content

Lin CH, Lo FS, Huang YY, et al. Evaluation of disease complications among adults with type 1 diabetes and a family history of type 2 diabetes in Taiwan. *JAMA Netw Open.* 2021;4(12):e2138775. doi:10.1001/jamanetworkopen.2021.38775

eTable. The ICD Coding of Diseases

eFigure 1. The Development of Comorbidities Over Time. (A) Hypertension. (B) Hyperlipidemia.

eFigure 2. The Development of Microvascular Complications Over Time. (A) Nephropathy. (B) Retinopathy. (C) Peripheral neuropathy.

eFigure 3. The Development of Macrovascular Complications Over Time. (A) MACEs (B) Stroke (C) CAD.

This supplemental material has been provided by the authors to give readers additional information about their work.

© 2021 Lin CH et al. *JAMA Network Open*.

eTable. The ICD Coding of Diseases

**Disease**	**ICD9 code, A code, or order_code**	**ICD10**	**Procedure Code**	**Source**
T1D	250.x1, 250.x3	E10		Catastrophic Illness Database
T2D	250.xx except 250.x1 or 250.x3 and using DM medications	E11		Two outpatient records or one inpatient records
Nephropathy	250.4	E11.21, E11.29, E10.22, E10.29, E11.65, E10.21		Two outpatient records or one inpatient records
Retinopathy	250.5	E08.3, E09.3, E10.3, E11.3, E13.3		Two outpatient records or one inpatient records
Peripheral neuropathy	250.6	E08.4, E09.4, E10.4, E11.4, E13.4		Two outpatient records or one inpatient records

© 2021 Lin CH et al. *JAMA Network Open*.

Stroke	430-437, A290-A294	I60、I61、I62、I65、I66、I67、G45、G46		One inpatient record
Hypertension	401, 402, A26	I10、I11		Two outpatient records or one inpatient records
Hyperlipidemia	272, A182	E78.4、E78.5		Two outpatient records or one inpatient records
Coronary artery disease<br>(CAD)	410	I21、I22		Two outpatient records or one inpatient records
MACE (major adverse cardiac events)				
Myocardial infarction	410	I21, I22		One inpatient record
Heart failure	428	I50		One inpatient record

© 2021 Lin CH et al. *JAMA Network Open*.

Stroke	430-437	I60, I61, I62, I65, I66, I67, G45, G46		One inpatient record
Malignant dysrhythmia	426.0, 426.12–426.13, 426.51–426.52, 426.54, 427.1, 427.4, 427.41–427.42, 427.5	I44, I45, I46, I47.2, I49.01, I49.02		One inpatient record
Cardiogenic shock	785.51	R57.0		One inpatient record
Percutaneous coronary intervention (PCI)			33076A, 33076B, 33077A, 33077B, 33078A, 33078B	One inpatient record
Coronary artery bypass surgery (CABS)			68023A, 68023B, 68024A, 68024B, 68025A, 68025B	One inpatient record
Thrombolysis therapy			B016526248, K000743248, K000744238	One inpatient record

© 2021 Lin CH et al. *JAMA Network Open*.

eFigure 1. The Development of Comorbidities Over Time. (A) Hypertension. (B) Hyperlipidemia.

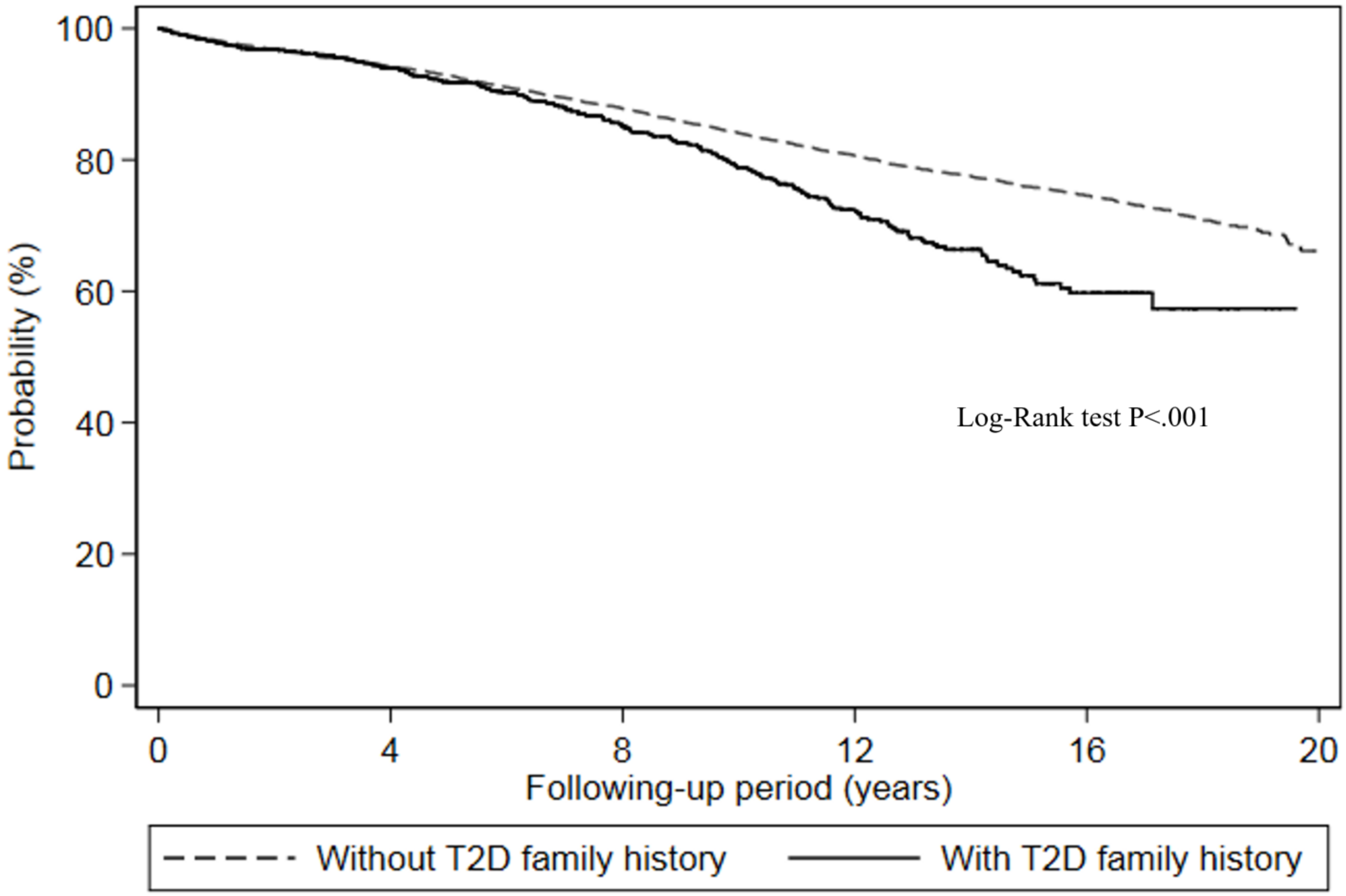

Figure 1 A).Hypertension

© 2021 Lin CH et al. *JAMA Network Open*.

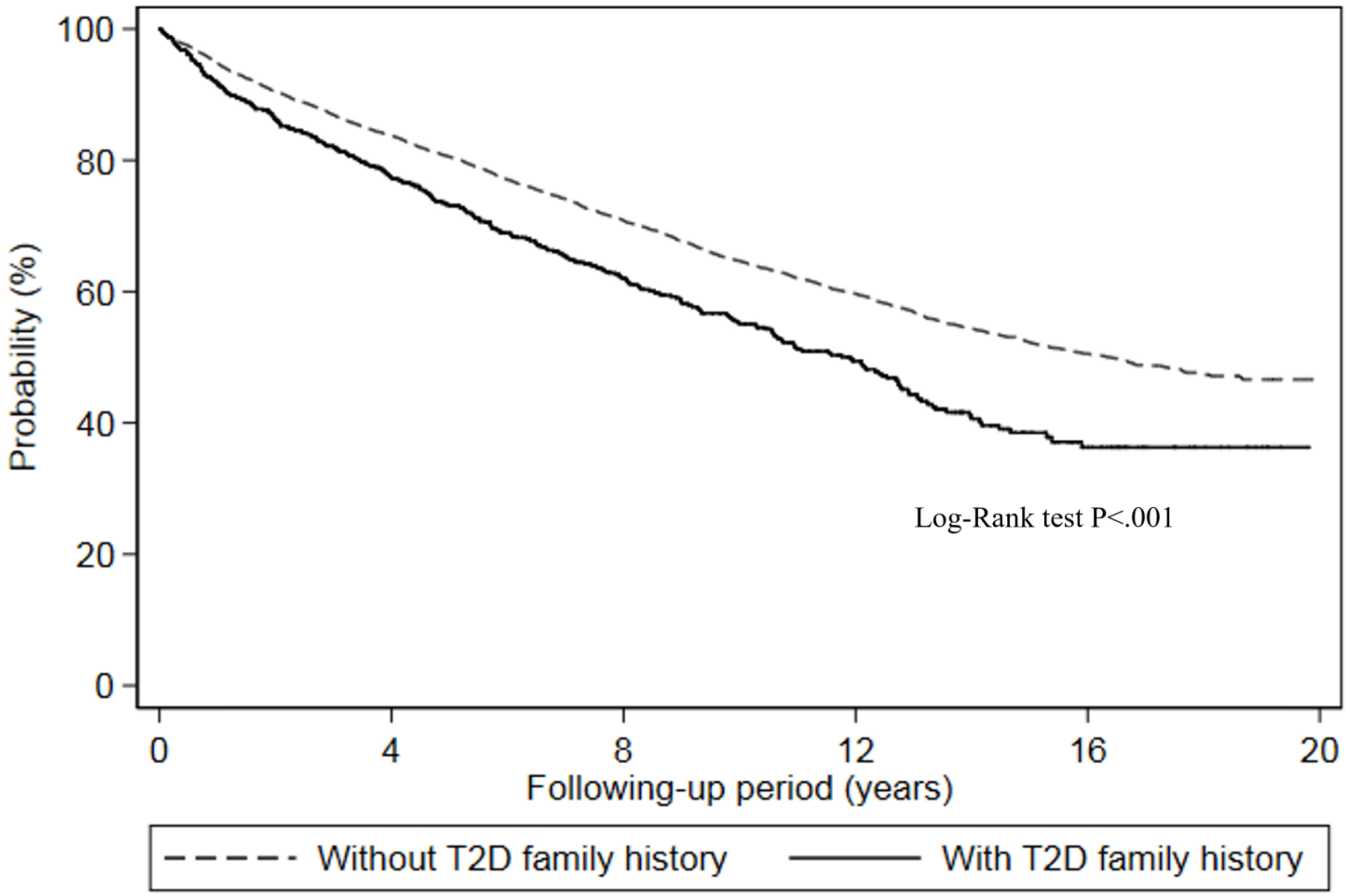

Figure 1 B).Hyperlipidemia

© 2021 Lin CH et al. *JAMA Network Open*.

eFigure 2. The Development of Microvascular Complications Over Time. (A) Nephropathy. (B) Retinopathy. (C) Peripheral neuropathy.

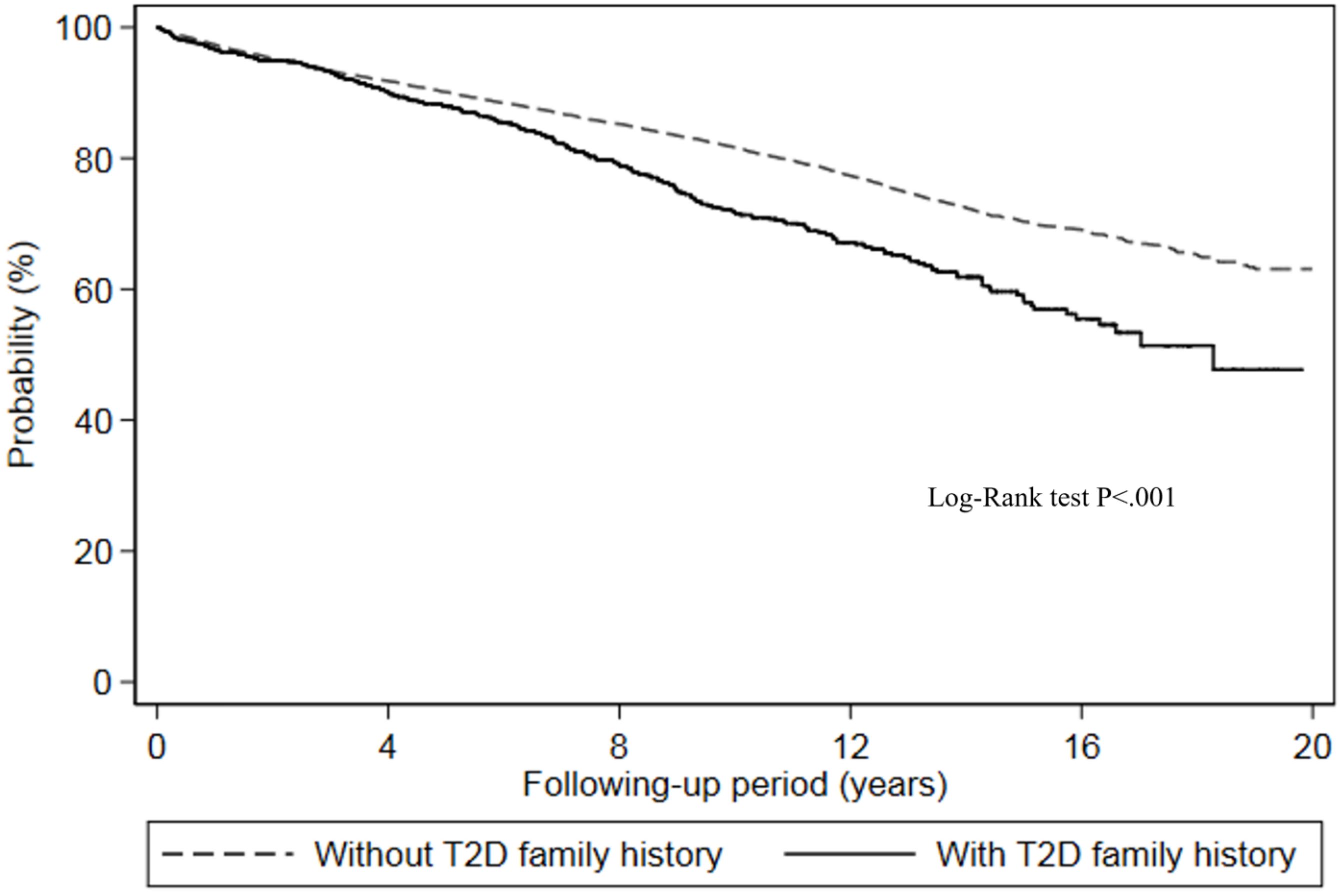

Figure 2 A).Nephropathy

© 2021 Lin CH et al. *JAMA Network Open*.

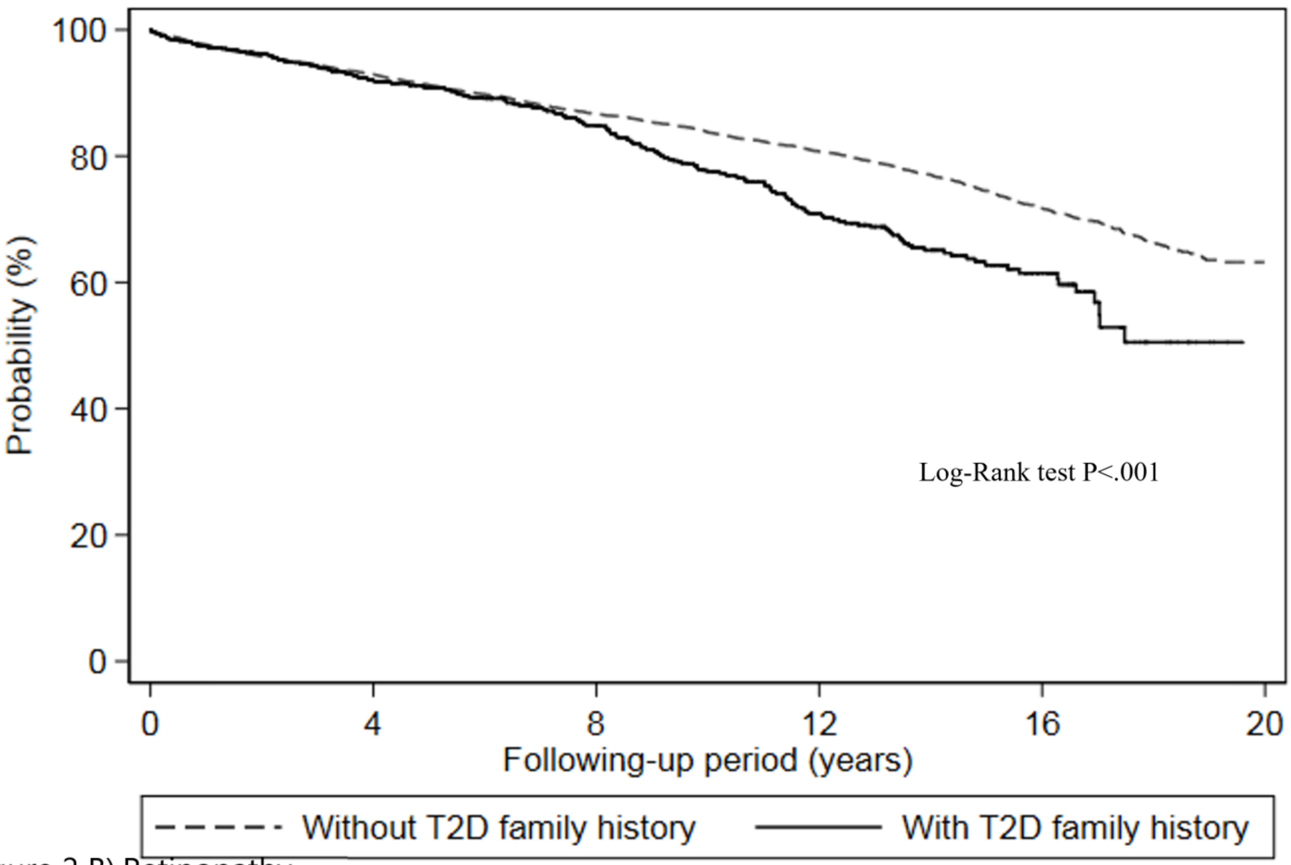

Figure 2 B).Retinopathy

© 2021 Lin CH et al. *JAMA Network Open*.

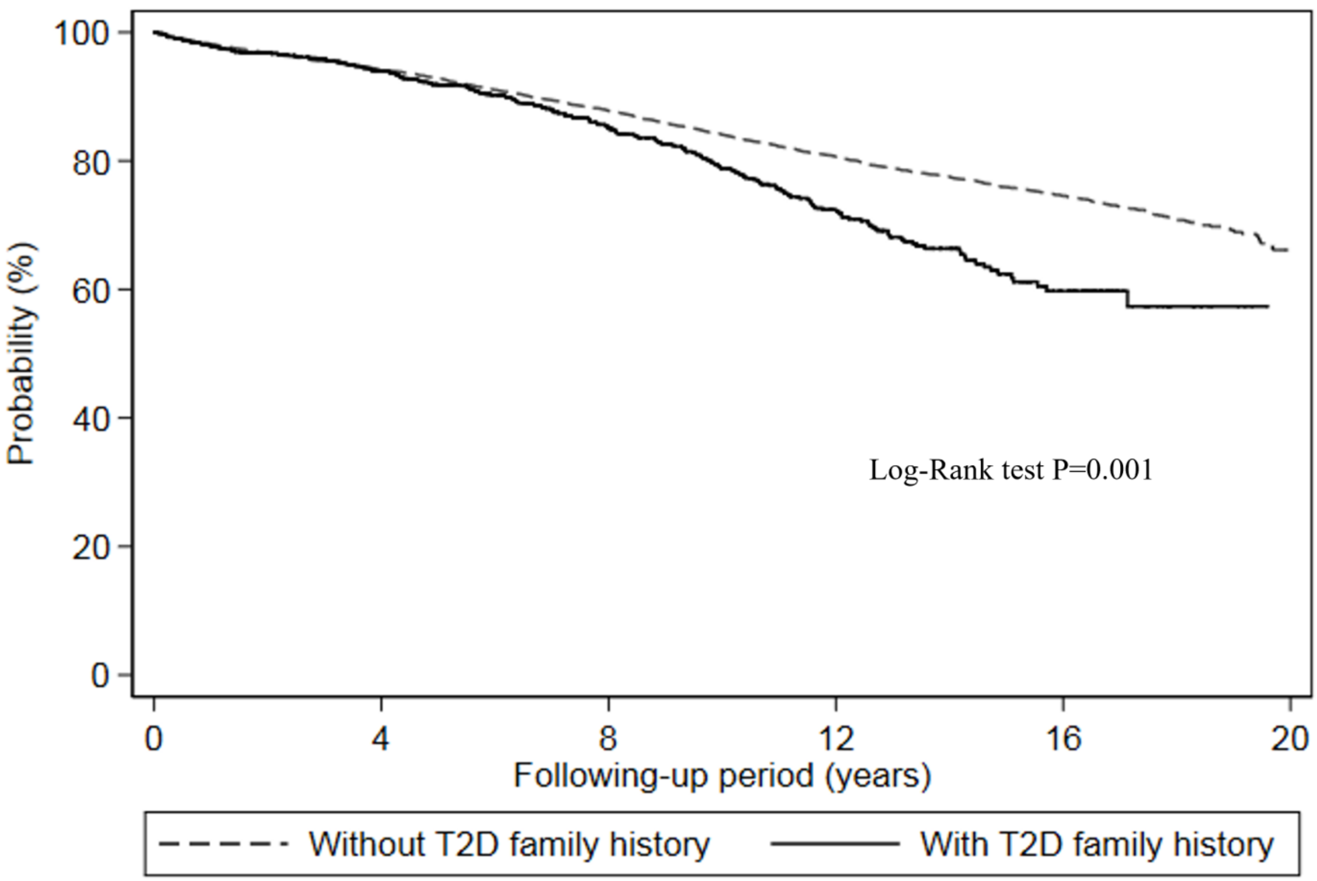

Figure 2 C).Peripheral neuropathy

© 2021 Lin CH et al. *JAMA Network Open*.

eFigure 3. The Development of Macrovascular Complications Over Time. (A) MACEs (B) Stroke (C) CAD.

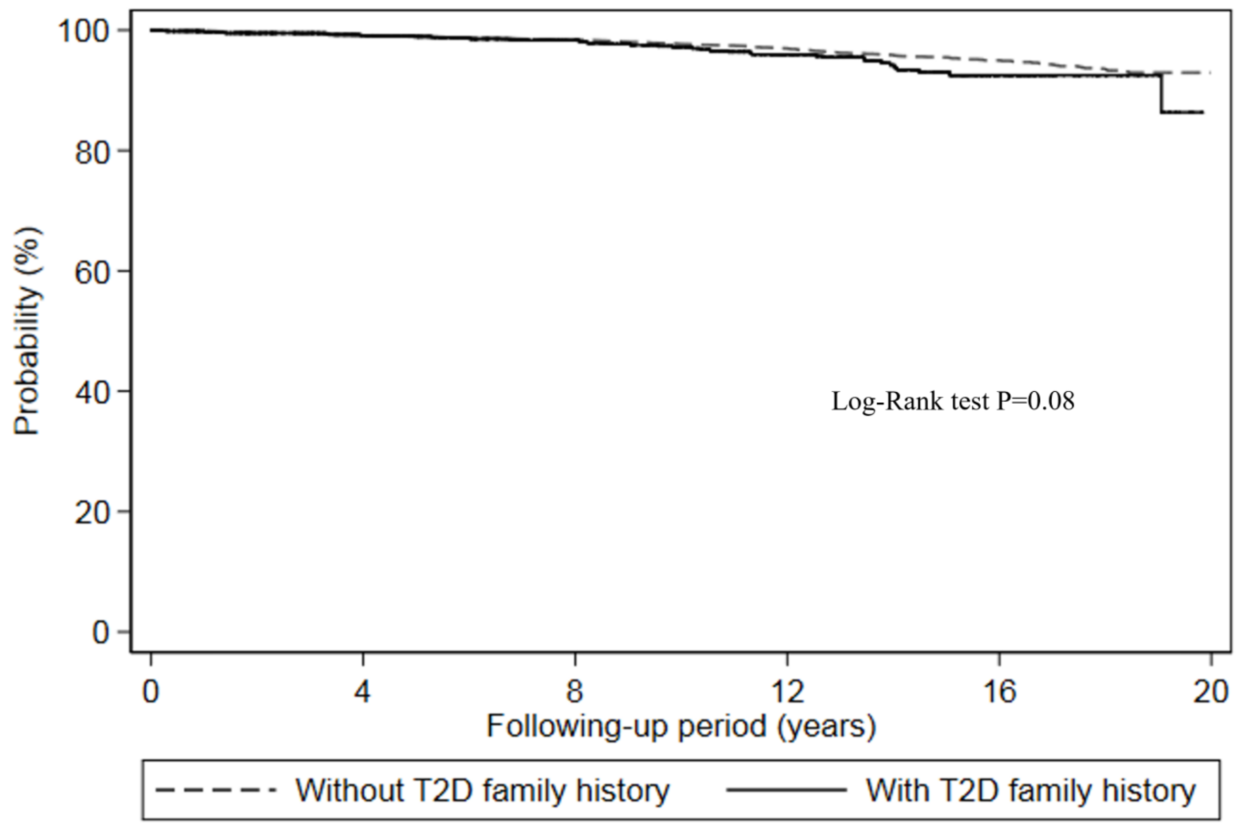

Figure 3 A).MACEs

© 2021 Lin CH et al. *JAMA Network Open*.

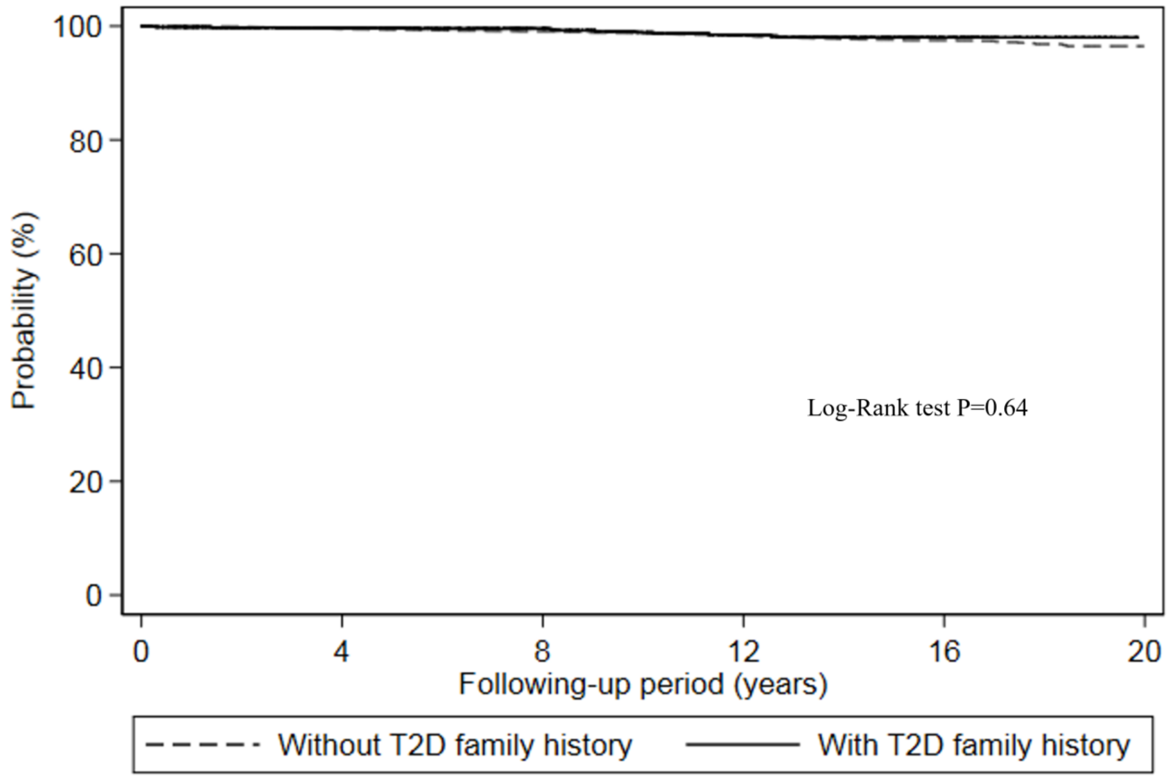

Figure 3 B).stroke

© 2021 Lin CH et al. *JAMA Network Open*.

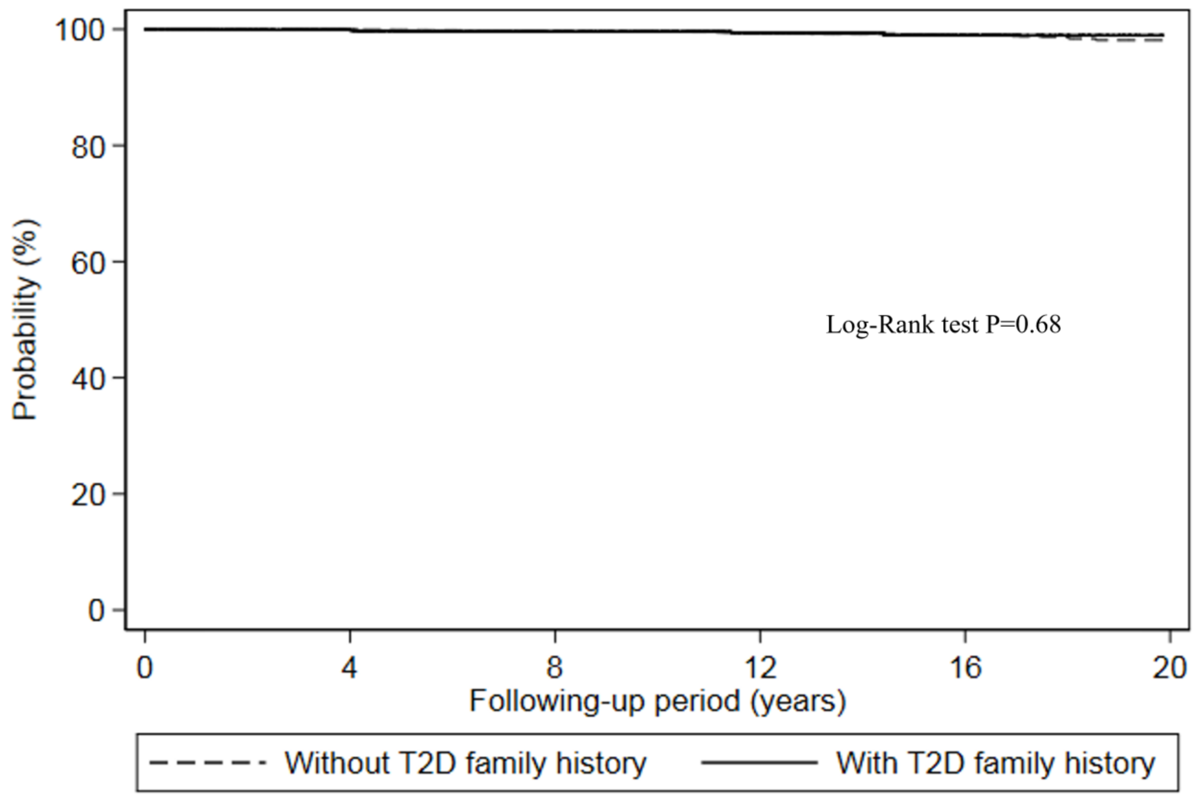

Figure 3C).CAD

© 2021 Lin CH et al. *JAMA Network Open*.
